# Quality by Design for Development, Optimization and Characterization of Brucine Ethosomal Gel for Skin Cancer Delivery

**DOI:** 10.3390/molecules26113454

**Published:** 2021-06-07

**Authors:** Tamer A. Ismail, Tamer M. Shehata, Dalia I. Mohamed, Heba S. Elsewedy, Wafaa E. Soliman

**Affiliations:** 1 Department of Clinical Laboratory Sciences, Turabah University College, Taif University, P.O. Box 11099, Taif 21944, Saudi Arabia; t.ismail@tu.edu.sa; 2 Department of Pharmaceutical Sciences, College of Clinical Pharmacy, King Faisal University, Al-Hofuf 36362, Saudi Arabia; helsewedy@kfu.edu.sa; 3 Department of Biochemistry, Zagazig Branch, Agriculture Research Center, Animal Health Research Institute, Zagazig 44519, Egypt; Daliaibrahim79@yahoo.com; 4 Department of Biomedical Sciences, College of Clinical Pharmacy, King Faisal University, Al-Hofuf 36362, Saudi Arabia; weahmed@kfu.edu.sa; 5 Department of Microbiology and Immunology, Faculty of Pharmacy, Delta University for Science and Technology, Gamasa, Mansoura 11152, Egypt

**Keywords:** brucine, ethosome, transdermal drug delivery, skin, optimization

## Abstract

Natural products have been extensively used for treating a wide variety of disorders. In recent times, Brucine (BRU) as one of the natural medications extracted from seeds of nux vomica, was investigated for its anticancer activity. As far as we know, this is the first study on BRU anticancer activity against skin cancer. Thus, the rational of this work was implemented to develop, optimize and characterize the anticancer activity of BRU loaded ethosomal gel. Basically, thin film hydration method was used to formulate BRU ethosomal preparations, by means of Central composite design (CCD), which were operated to construct (3^2^) factorial design. Two independent variables were designated (phospholipid percentage and ethanol percentage) with three responses (vesicular size, encapsulation efficiency and flux). Based on the desirability function, one formula was selected and incorporated into HPMC gel base to develop BRU loaded ethosomal gel. The fabricated gel was assessed for all physical characterization. In-vitro release investigation, ex-vivo permeation and MTT calorimetric assay were performed. BRU loaded ethosomal gel exhibited acceptable values for the characterization parameters which stand proper for topical application. In-vitro release investigation was efficiently prolonged for 6 h. The flux from BRU loaded ethosome was enhanced screening optimum SSTF value. Finally, in-vitro cytotoxicity study proved that BRU loaded ethosomal gel significantly improved the anticancer activity of the drug against A375 human melanoma cell lines. Substantially, the investigation proposed a strong motivation for further study of the lately developed BRU loaded ethosomal gel as a prospective therapeutic strategy for melanoma treatment.

## 1. Introduction

Nanotechnology is a discipline involves the design, development, characterization and application of nanoscale carrier systems in different aspects of nanomedicine [1]. It compromises various techniques in treatment, especially in the case of cancer therapy in order to increase drug efficacy, selectivity and support the transporting of poorly water soluble drugs [2,3]. Skin cancer is a form of malignancy that is extensively spread in many countries. In certain cases, the most appropriate strategy for skin cancer treatment is through transdermal delivery since it would deliver higher concentrations of the medications to the target site directly [4].

In recent times, transdermal drug delivery systems have gained much attention owing to their high competence factors, such as more patient compliance, low frequency of dosing and avoiding many other problems usually related to the conventional oral dosage form [5]. Several dosage forms fall under the category of transdermal drug delivery systems, encompassing a variety of dosage forms such as gel, emulgel, nanoemulgel and patches [6]. 

One of the key restrictions in delivering the medication via the skin is the low permeability of these drugs through the skin barrier, which could result in lower transdermal flux. As a result, enhancement of drug penetration became a necessary object that could be achieved via different strategies including the use of penetration enhancers. Ethanol is one of the most common enhancers that have the capability to advance the passage of the medication through the skin and facilitate the percutaneous diffusion. Relatively modern topical drug delivery systems incorporating ethanol have been developed, such as ethosome [7]. Several literatures had shown the effectiveness of a ethosomal carrier over other a nanocarrier which propose its substantial influence in drug delivery systems [8]. 

Ethosome has been identified as a superior drug delivery carrier in improving drug skin penetration. It principally started as a vesicular carrier with elastic membrane that improve the solubility of the materials and facilitated its incorporation. It was composed of phospholipid, cholesterol, water and ethanol [9]. Ethosome possesses bilayer, aqueous and lipid layers; thus, it could incorporate both hydrophilic and hydrophobic drugs. Ethosome can be fabricated easily without any complicated manufacturing protocol [10] and provides higher stability and solubility for the incorporated therapeutic agent [11]. Moreover, it could improve the pharmacokinetic parameters and enhance the drug efficacy and increase the therapeutic window [12,13].

BRU has been widely used as a natural medication which is the main constituent of Strychnos nux-vomica seed [14]. Several uses were reported for BRU as an analgesic [15] with anti-inflammatory [16], anti-proliferative and antitumor activity [17,18]. In order to enhance BRU efficacy and overcome its low solubility, several prospects were studied to develop new formulations incorporating BRU [19]. Based on that, it was motivating to develop innovative appropriate formulations like ethosome to deliver BRU with least drawbacks [20]. Consequently, to reach more optimized formulation, quality by design (QbD) concept has been applied using different methodologies to obtain the best quality design [21].

QbD is a systematic organized process that is concerned with the quality of the pharmaceutical product. It proceeds by running specific key factors signifying the independent variables, and examining their influence on the dependent observed responses [22]. In other words, QbD offers and executes a perfect outline for the model to be designed and to meet the desired standards [23]. Response surface methodology (RSM) is a tool that produces large amount of data from the least work. It correlates with Central Composite Design (CCD) which is one of the most prevalent software that is based on specific mathematical, statistical equations and certain graphs for modeling the design [24,25]. 

In these contexts, BRU loaded into different ethosomal formulations was prepared, followed by characterization and optimization using CCD. The optimized formula was loaded into a gel formulation that could be positively applied to the skin. BRU loaded ethosomal gel was characterized as a topical preparation for its physical properties and in-vitro and ex-vivo permeation studies. Ultimately, it was examined for its in-vivo antitumor activity and compared to free drug, blank ethosome and conventional gels.

## 2. Results

### 2.1. Experimental Design for the Ethosomal Formulations

#### Statistical Analysis of the Data

Twelve experiments were run via CCD software attempting to optimize the fabricated ethosomal formulations and record the influence of the independent variables on the dependent response as displayed in Table 1. As known for *p*-value, if being less than 0.05, this confirms a statistically significant model [26]. According to that, it was apparent that the best-fit model compared to the other models was the linear one for all responses. As shown in Table 2, *p*-value < 0.0001 was detected for all independent variables in all responses, a matter that confirms the significant effect of these variables on the studied response. On the other hand, higher F-value is more desirable as per exhibited for all responses in our study that indicate less error in the model. Additionally, it was signified that F-value of the 3 models was 42.04, 42.74 and 160.61 for Y_1_, Y_2_ and Y_3_, respectively, which revealed a significant model. Furthermore, non-significant lack of fit is mandatory to fit the model, and as shown in the observed responses Y_1_, Y_2_ and Y_3_, it was 2.43, 1.81 and 1.08 with relative *p*-value 0.2489, 0.3349 and 0.5160, respectively, which considered good non-significant lack of fit [27]. 

### 2.2. Vesicular Size and Size Distribution (PDI) Determination

**Figure 1:**
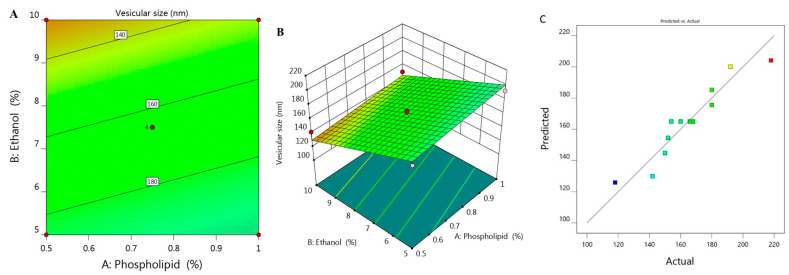
(**A**) 2D contour graph, (**B**) 3D response surface plot demonstrating the influence of independent variables (X_1_) and (X_2_) on ethosome vesicular size (Y_1_) and (**C**) linear correlation plot between actual and predicted values for response (Y_1_).

Vesicular size of the prepared ethosome and their corresponding PDI values was estimated and found to be ranged between 118 ± 1.5 to 218 ± 3.0 with PDI 0.214 and 0.336 respectively as was illustrated previously in Table 1. It was shown that upon using constant percentage of ethanol X_2_ (5%) and increasing phospholipid concentration X_1_ from 0.5 to 1%, the vesicular size Y_1_ increased from 180 to 192 nm for F6 and F7, respectively. The same result was observed upon using 7.5% X_2_ and increasing X_1_ from 0.39 to 1.1%, Y_1_ would increase from 152 to 180 nm for F1 and F11, respectively. Similar findings were detected upon using 10% X_2_, increment in Y_1_ was observed from 142 to 150 nm for F2 and F5, respectively. This indicates the direct correlation between phospholipid concentration and the size of the ethosomal vesicle upon using fixed ethanol concentration. This finding could be explained on the basics of phospholipid function that construct lipid layers around the ethosome, which result in increasing the size. On the other hand, it was noticed that upon increasing X_2_ from 3.96 to 11%, a subsequent decrease in the ethosomal vesicular size from 218 to 118 nm for F12 and F10, respectively, was recorded. A reverse action was observed for ethanol concentration variable X_2_ which points toward decreasing the vesicular size because of the great stabilization provided to the ethosome that resulted in smaller vesicular size [28]. The influence of both independent variables X_1_ and X_2_ on the BRU loaded ethosome vesicular size (Y_1_) is visibly explained through 2D contour and 3D-response surface plot as displayed in Figure 1A,B.

The influence of these variables was further emphasized using the following regression equation that revealed the positive effect of variable X_1_ in addition to the negative action related to variable X_2_ on Y_1_:

Y_1_ = 225.68 + 29.79 X_1_ − 11.071 X_2_ (1)

Moreover, the linear correlation between the predicted versus the actual responses illuminating that the predicted R^2^ for Y_1_ is (0.8052) which was in realistic agreement with the adjusted one (0.8818) as described in Table 2. The residual value is apparent as dispersed between the two sides of the line suggesting a reasonable correlation among that experimental data and the predicted value as portrayed in Figure 1C. Also, the ability of the system to suggest the model is enhanced since the R^2^ value is near to one (0.9033) in addition to the anticipated adequate precision that was 17.707 indicating that the model could navigate the design space. 

### 2.3. Encapsulation Efficiency (EE)

The percentage of BRU encapsulated into the developed ethosome was calculated and represented as % of EE that ranged between 50.2 ± 1.8 and 77 ± 1.2% as presented in Table 1. It was observed that increasing concentration of X_1_ from 0.5 to 1% while keeping X_2_ constant (5%), the EE increased from 55.8 to 58.9% for F6 and F7 respectively. On the same track, on increasing X_1_ from 0.39 to 1.1% while maintaining X_2_ concentration (7.5%), the EE raised from 61.4 to 73.5% for F1 and F11, respectively. There was a similar result upon increasing X_1_ from 0.5 to 1% while keeping X_2_ concentration (10%), the EE increased from 74 to 76.3% for F5 and F10, respectively. From the previous data, it could be concluded that there was a progressive influence of phospholipid on the EE of the obtained ethosome. This could be ascribed to the vesicular size, where the larger the vesicular size, the greater would be the space that could entrap large amount of BRU, which could provide higher percentage of EE as a result [29]. Likewise, greater phospholipid concentration could build multilayer that have the ability to entrap more BRU within the layers which could enhance the EE as well [30]. Moreover, cholesterol plays a crucial role in improving the EE due to its structure as a rigid steroid, hence a lower permeability of the vesicles and hindering of the leakage [31]. As for ethanol concentration, the same positive effect of phospholipid was detected, since the higher the concentration of ethanol, the better the ethosomal EE. This is most likely due to improvement of the drug solubility which helps in increasing the encapsulation of the drug inside the formulation [32]. 

The derived polynomial equation for that investigated factor (Y_2_) that clarifies its relation with all responses is given as:

Y_2_ = 30.945 + 11.256 X_1_ + 3.675 X_2_ (2)

**Figure 2:**
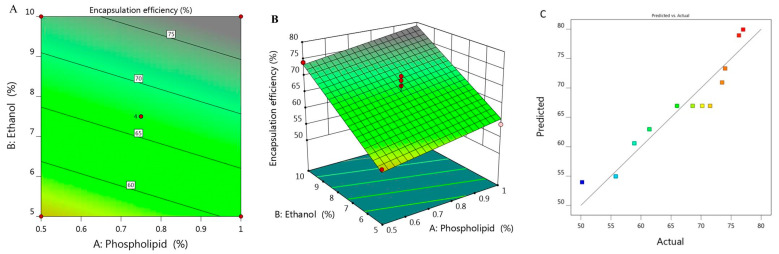
(**A**) 2D contour graph, (**B**) 3D response surface plot demonstrating the influence of independent variables (X_1_) and (X_2_) on encapsulation efficiency (Y_2_) and (**C**) linear correlation plot between actual and predicted values for response (Y_2_).

It was noticed that both responses X_1_ and X_2_ provided significant model terms. Additionally, as displayed in Table 2, the adjusted R^2^ signified value of 0.8836 which was concomitant with the predicted R^2^ (0.8310) which probably affirms the linearity of the data as further confirmed in Figure 2C. Alongside, the value of R^2^ was recorded (0.9047) and the required adequate precision of 17.68 which indicate the capability of the model to direct the design space. Figure 2A,B displayed the 2D contour and 3D response surface plot of EE that emphasized the positive impact of all variables Y_1_, Y_2_ and Y_3_. 

### 2.4. Ex-Vivo Study (Skin Permeation Study)

**Figure 3:**
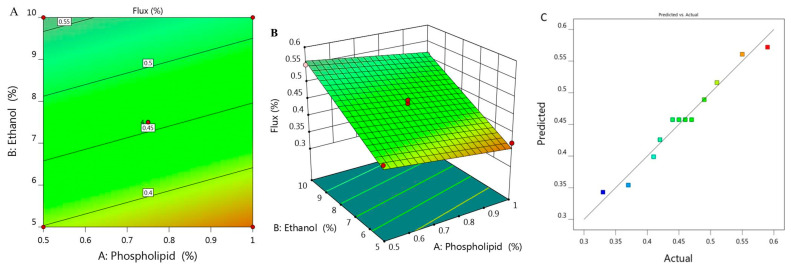
(**A**) 2D contour graph, (**B**) 3D response surface plot demonstrating the influence of independent variables (X_1_) and (X_2_) on the flux (Y_3_) and (**C**) linear correlation plot between actual and predicted values for response (Y_3_).

Study of skin permeation was implemented and permeation parameters with the permeation profile of all examined formulations are displayed in Figure 3 and Table 1. SSTF value of all ethosomal formulations were ranged between 0.33 ± 0.015 to 0.59 ± 0.04 µg/cm^2^·h. It was remarkable that along with increasing X_1_ from 0.5 to 1% while keeping concentration of X_2_ (5%) constant, the flux from ethosome would decrease from 0.41 to 0.37 µg/cm^2^·h for F6 and F7, respectively. Likewise, upon using 7.5% X_2_ and increasing X_1_ from 0.39 to 1.1, the flux decreased from 0.49 to 0.42 µg/cm^2^·h for F1 and F11 respectively. Similarly, increasing X_1_ from 0.5 to 1% upon using 10% X_2_ resulted in lowering in flux from 0.55 to 0.51 µg/cm^2^·h for F2 and F5, respectively. In light of the previous findings, it was stated that increasing the concentration of phospholipid while keeping concentration of ethanol constant would result in a negative effect as it decreased the value of SSTF. However, it was obvious that increment in concentration of X_2_ from 3.96 to 11% provided enhancement in the percentage of flux from 0.33 to 0.59 µg/cm^2^·h. Definitely, this indicated synergistic effect of ethanol on the drug permeation across the skin, which could be returned to its effective role as a penetration enhancer [33]. In addition to the lipid-softening effect of ethanol that facilitate the penetration of the drug and improve the permeation as well [34]. In view of this, the skin permeation is affected by varying the concentration of phospholipid and ethanol [35,36]. 

Figure 3A,B depict 2D contour graph and 3D surface plot that provide interpretation for the variable effect on the value of flux response. Further, as detailed in Table 2, the predicted R^2^ was 0.9483, which revealed a reasonable correspondence with the adjusted R^2^ (0.9667) in addition to the proper value of R^2^ (0.9727) and adequate precision (34.55) that represent adequate signal. The following is the regression equation that clarifies the role of the variables on the flux:

Y_3_ = 0.2817 − 0.0894 X_1_ + 0.0323 X_2_ (3)

### 2.5. Optimizing the Independent Variables

**Figure 4:**
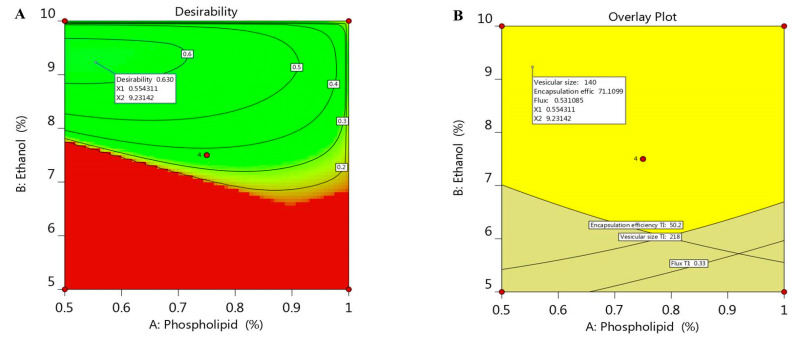
(**A**) Optimization plot screening the effect of different variables X_1_ and X_2_ on overall desirability and (**B**) overlay plot of the desired responses for the optimal region of ethosome formulation.

Optimization process is aimed to adopt the optimum constraints to reach the utmost desirability and get ethosomal formulation with proper quality features [37]. A numerical optimization was processed via the desirability function depending on the resultant data that were obtained from various graphs drawn by the design software [38]. The goal in optimizing the ethosome was dependent on assigning the independent variables toward certain parameters; namely, to minimize both phospholipid and ethanol concentration in addition to adjusting the responses as to minimize the vesicular size and maximize both the EE and the flux. The selected formula was easily predicted using point prediction option in the software. As shown in Table 3, 0.55% phospholipid and 9.2% ethanol were the predicted independent variables that was expected by CCD; however, the predicted response values were 140 nm for the vesicular size, 71.1% EE and 0.531 µg/cm^2^·h for the flux of the drug from the skin membrane. The previous predicted values for response suggested the maximum desirability value (0.603) as seen in Figure 4A, in addition to the overlay plot that labels the zone at which the optimized criterion is met (Figure 4B). The previous suggested values of the independent variables were used to develop the optimized ethosome and compare the result of its characterization with the predicted response. The obtained experimental value of responses were vesicular size of (145.6 nm), EE (72.9%) and Flux of (0.513 µg/cm^2^·h), which proved to be very close to that of the predicted values proposed by the CCD system.

### 2.6. Vesicular Size, PDI and Zeta Potential of Optimized BRU Loaded Ethosomal Formulation

**Figure 5:**
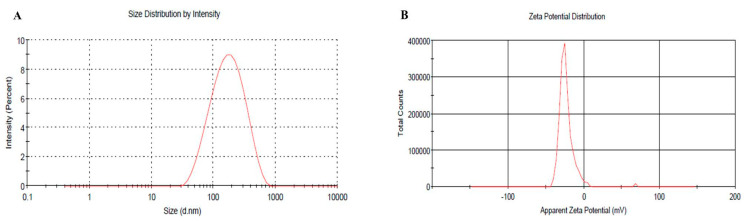
(**A**) Vesicular size distribution curve (**B**) Zeta potential of optimized BRU loaded ethosome.

The vesicular size of the optimized BRU loaded ethosomal formulation was assessed (145.6 nm) with good PDI value (0.259) and the related distribution curve was presented in Figure 5A. Concerning the ethosomal surface charge, zeta potential was estimated and it showed a charge of −23.3 ± 8.2 mV as revealed in Figure 5B. In fact, the presence of ethanol in the preparation shifted the charge toward negative that could successfully enhance the electrostatic repulsion and inhibit aggregation of vesicles which would improve the stability of the formulation.

According to the obtained results in the previous investigations, HPMC gel base was fabricated and mixed with the optimized BRU loaded ethosome in order to attain BRU loaded ethosomal gel formulation. The ethosomal gel is more applicable for skin treatment and was subjected to further evaluations to be compared with conventional BRU loaded gel. 

### 2.7. Characterization of the Developed BRU Loaded Ethosomal Gel

Table 4 displays the different characters that were evaluated for the developed gel and ethosomal gel. Physical examination of BRU loaded gel and ethosomal gel certified homogeneity, smoothness and the acceptable physical appearance of the formulations. In order to avoid any skin irritation upon application, pH measurement confirmed that the values were satisfactory. Furthermore, viscosity and spreadability results revealed reasonable data that being adequate for skin application.

### 2.8. Morphological Evaluation

**Figure 6:**
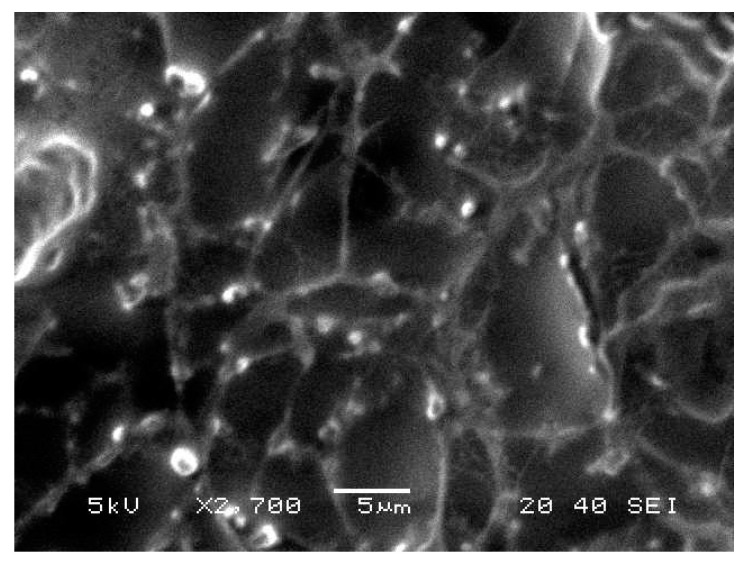
Scanning electron microscopy of BRU loaded ethosomal gel formulation.

BRU loaded ethosomal gel was evaluated for its morphological characterization via scanning electron microscopy as displayed in Figure 6. It was apparent that vesicles were spherical with smooth surfaces.

### 2.9. In Vitro Release Experiment

**Figure 7:**
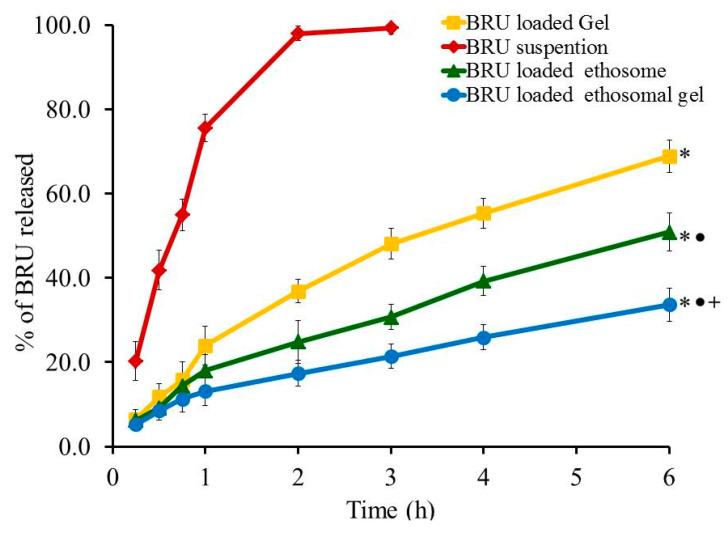
Profile of In vitro release experiment from BRU preparations at 37 °C. Data are expressed as the mean ± SD (*n* = 3). * *p* < 0.05 comparable to BRU suspension, ● *p* < 0.05 comparable to BRU loaded gel and + *p* < 0.05 comparable to BRU loaded ethosome.

Profile of in-vitro release experiment was designed and the percentage of BRU released from all developed formulations, as well as from BRU suspension, is portrayed in Figure 7. The percentage of BRU released from BRU loaded gel, BRU loaded ethosome and BRU loaded ethosomal gel was 68.87 ± 3.9, 50.87 ± 4.5 and 33.67 ± 3.92% respectively over a period of 6 h. It was apparent that the percentage of BRU released from loaded gel is significantly greater than that released from ethosome and ethosomal gel (*p* < 0.05). This could be accredited to the gel composition that includes higher aqueous content that could speed up the transfer of BRU out of the gel in to the release media. Moreover, the percentage of BRU released from ethosome was significantly larger than that released from ethosomal gel (*p* < 0.05). Actually, viscosity of the formulation plays a vital role in the release study, hence ethosomal formulation being less viscous than ethosomal gel that would facilitate the transport of the entrapped BRU to the dissolution media [39].

### 2.10. Ex-Vivo Investigation

**Figure 8:**
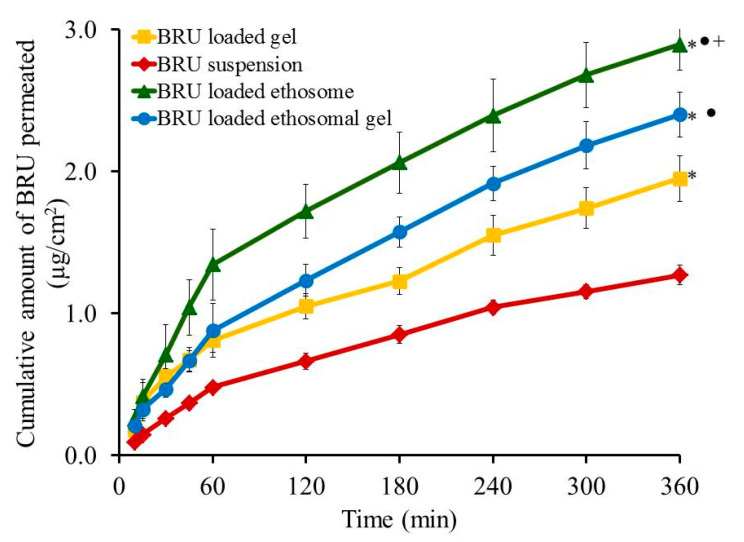
Outline skin permeation study of BRU from developed formulations. Data are expressed as mean ± SD (*n* = 3). * *p* < 0.05 comparable to BRU suspension, ● *p* < 0.05 comparable to BRU loaded gel and + *p* < 0.05 comparable to BRU loaded ethosomal gel.

Skin permeation investigation across skin rat was executed and the permeation pattern together with specific ex-vivo parameters was depicted in Figure 8 and Table 5. It was noticed that the amount of BRU permeated through skin membrane after 360 min from BRU loaded ethosome was 2.89 ± 0.18 µg/cm^2^, which is significantly larger than that permeated from BRU loaded ethosomal gel (2.4 ± 0.16 µg/cm^2^), BRU loaded gel (1.95 ± 0.16 µg/cm^2^) and BRU suspension (1.27 ± 0.07 µg/cm^2^) (*p* < 0.05). As a result, it was detected that the permeation from BRU loaded ethosome was enhanced by 2.42 ± 0.12 folds showing optimal SSTF (0.513 ± 0.03 µg/cm^2^·h) which is significantly higher than that from other formulations in the study (*p* < 0.05). On the other side, permeation from ethosomal gel formulation was enhanced by 1.89 ± 0.12 folds displaying SSTF value 0.4 ± 0.03 µg/cm^2^·h which is significantly less than that of ethosome itself and greater than the values of gel formulation that improve the permeation by approximately 1.53 ± 0.126 folds with SSTF value 0.325 ± 0.027. As a matter of fact, lower permeation from BRU loaded gel was due to its colloidal nature [40]. However, higher values corresponding to BRU loaded ethosome is returned to its lower viscosity than BRU loaded ethosomal gel that resulted in higher release and higher permeation as well [13,41].

### 2.11. In-Vitro Cytotoxicity

**Figure 9:**
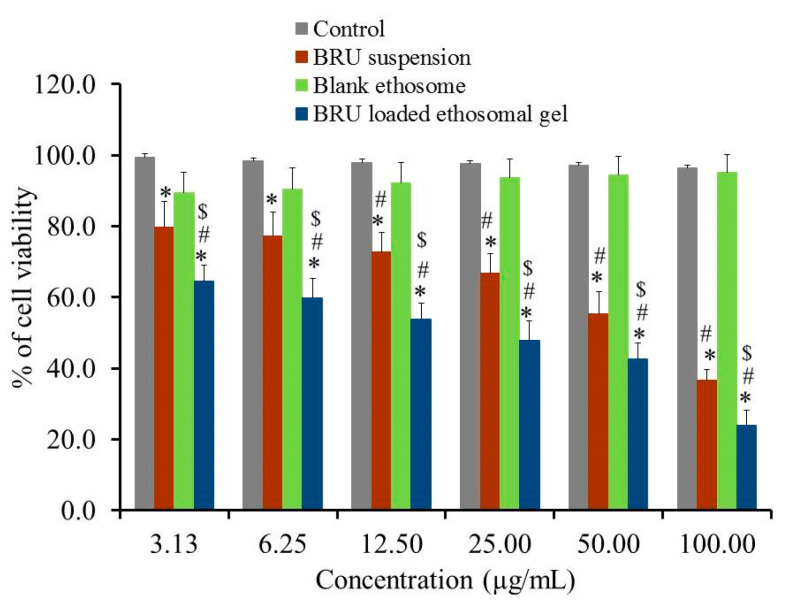
In-vitro cytotoxicity study of BRU suspension. Blank ethosome and BRU loaded ethosomal gel against A 375 cell line for 48 h. Data are expressed as mean ± SD (*n* = 3). * *p* < 0.05 comparable to control, # *p* < 0.05 comparable to blank ethosome and $ *p* < 0.05 comparable to BRU suspension.

Evaluating cytotoxicity of the developed BRU ethosomal formulation was a very important factor in determining the possibility of applying such a nanocarrier in skin cancer treatment. In view of that MTT colorimetric assay was investigated for BRU suspension, blank ethosome and BRU loaded ethosomal gel against A375 cell line. As apparent in Figure 9, percentage of cell viability was significantly lessened for BRU loaded ethosomal gel achieving lower IC50 values of 29.91 ± 5.59 µg/mL if compared to that of BRU suspension exhibiting IC50 value 65.96 ± 9.7 µg/mL (*p* < 0.05). The reduced cell viability upon treating with BRU loaded ethosomal gel could be attributed to the sustained release of BRU from ethosome which result in existence of the drug in contact with the tumor cells providing greater anticancer effect [42]. Additionally, it was noteworthy that including the drug within the ethosomal formulation would ameliorate its cytotoxic effect more than the free drug [43]. However, treating the cancer cells with these formulations, BRU suspension and BRU loaded ethosomal gel, showed a concentration dependent cytotoxicity [44]. On the other hand, higher percentage of cell viability was demonstrated in case of blank ethosome that could reach more than 95% indicating that it does not have any cytotoxic effect against cancer cells. This actually proved the prominence of the ethosomal gel as a carrier for the drug [45].

## 3. Materials

BRU was supplied from Alpha Chemika, (Mumbai, India). Ethanol, soy lecithin, cholesterol and hydroxy propyl methyl cellulose (HPMC K15M) were purchased from Sigma Aldrich (St. Louis, MO, USA). Fetal bovine serum (FBS) was supplied from Sigma Aldrich (St. Louis, Mo, USA). Tetrazolium dye (MTT reagent) was procured from Loba Chemie (Mumbai, India). All other chemicals of analytical grade were obtained from Sigma, (St. Louis, MO, USA).

### 3.1. Experimental Design Study

A two factor, three level (32) factorial design was developed via (RSM) using Design-Expert version 12.0 software (Stat-Ease, Minneapolis, MN, USA). Fundamentally, sequences of preliminary studies were executed in order to determine the main factors of the investigation. Accordingly, phospholipid percentage (X_1_) and Ethanol percentage (X_2_) were nominated to represent the independent variables that were inspected for their effects on the vesicular size (Y_1_), encapsulation efficiency (Y_2_) and flux (Y_3_) of the developed ethosome. Data in Table 6 demonstrated two independent variables that showed their responses on the dependent variables (Y_1_), (Y_2_) and (Y_3_) using three different levels (−1, 0, 1). To check the statistical analysis of the data and the designed model, Analysis of variance (ANOVA) was implemented. Next, certain graphs were plotted such as 2D Contour and 3D-response surface in addition to mathematical equations for the response which help to illustrate the relationship between the data as follow:

Y = bo + b1X_1_ + b2X_2_ + b12X_1_X_2_ + b11X_2_ + b22X_2_ (4)

In which Y signifies the dependent variable whereas b0 symbolizes the intercept; b1, b2, b12, b11 and b22 are the regression coefficients. X_1_ and X_2_ represent the main factors; X_1_X_2_ represents the interactions between main factors and X_12_, and X_22_ specify the polynomial terms.

### 3.2. Preparation of BRU Loaded Ethosome

BRU loaded ethosome was prepared by thin film hydration method that reported previously by Sakdiset et al. [28]. Concisely, BRU (50 mg) was used in addition to same amount of cholesterol and both were added into clean, desiccated round bottomed flask. The quantified amount of phospholipid (Table 1) was added to the flask and the mixture was dissolved in ethanol. Afterward, ethanol was evaporated using rotary evaporator (Heidolph, GmbH, Co, KG, Germany) to form a thin lipid film at the internal wall of the flask by allowing rotation at 100 rpm and heating up to 60 °C. The thin film was then hydrated using 10 mL hydroalcoholic solution (phosphate buffer pH 7.4 and ethanol) and kept at room temperature for 1 h to attain the final dispersion. To get a suitable vesicular size, BRU dispersion was sonicated using probe sonicator (XL-2000, Qsonica, Newtown, CT, USA) for 30 s at 150 watt. 

### 3.3. Characterization of Ethosomal Formulations

#### 3.3.1. Vesicular Size, Polydispersibility Index (PDI) and Zeta Potential Measurement

Vesicular size and PDI for all BRU loaded ethosome were examined measuring their dynamic light scattering at 25 °C and a scattering angle of 90° [46]. Zeta potential determination was assessed for the optimized BRU ethosomal gel in which the formulation surface charge was measured through the electrophoretic mobility using Zetasizer apparatus (Malvern Instruments Ltd., Worcestershire, UK) [47]. 

#### 3.3.2. Encapsulation Efficiency (EE)

The percentage of BRU encapsulated within the ethosomal system was estimated using centrifugation method. Centrifugation was permitted for 30 min at 30,000 rpm operating ultracentrifuge (Hitachi micro ultracentrifuge CS-FNX 120). At that point, the free drug was analyzed in the supernatant spectrophotometrically at λ_max_ 264 nm by means of spectrophotometer (U.V. Spectrophotometer, JENWAY 6305) [48]. The following equation would help in calculating the percentage of EE:

EE% = (T − F)/T(5)

whereas, T is the total quantity of BRU in the ethosome and F is the quantity of free drug.

### 3.4. Ex-Vivo Investigation

#### 3.4.1. Preparing Animal Skin

In order to investigate the permeation study, rat skin was selected as it is easily accessible, economically cheap and very comparable to human skin. Basically, by means of an electric clipper, the abdominal region of a male Wistar rat was shaved. Then, the rat skin was removed after scarifying the animals using inhaled chloroform. Until further studies, the detached skin was preserved at freezer [49]. 

#### 3.4.2. Skin Permeation Study

Since the amount of drug permeated through the skin reflects its activity a modified Franz diffusion cells was prepared in our lab in order to determine the amount of drug permeated across the rat skin [50,51,52]. The skin membrane was attached to a glass tube of permeation area 4.91 cm^2^ from one end and suspended into the receptor media containing phosphate buffer pH 7.4 (100 mL) and sodium azide (0.02%) at 37 ± 0.5 °C. Ethosomal formulation (0.5 mL) was added into the donor area and the tubes were protected from media evaporation by Parafilm (Bemis, Oshkosh, WI, USA) and allowed to stir at 100 rpm [53]. At different time intervals and up to 6 h, 1 mL of the sample was taken, measured spectrophotometrically at 264 nm using UV spectrophotometer (Jenway 6305 UV/Visible, Staffordshire, UK) and substituted with equivalent amount of fresh vehicle for tolerating the sink conditions [51]. Steady state transdermal flux (SSTF) and enhancement ratio (ER) were calculated for all formulations where they represent permeation parameters related to ex vivo study. 

SSTF indicates the amount of permeated drug/(area × time); and ER denotes SSTF-test/SSTF-control.

### 3.5. Incorporation of Optimized Ethosomal Formulation into HPMC Gel

The optimized BRU loaded ethosome was mixed with gel base prepared from HPMC (4% *w*/*w*). Simply, the required amount of the gelling agent was sprinkled slowly in a distilled water and kept on a magnetic stirrer (Jeio Tech TM-14SB, Medline Scientific, Oxfordshire, UK) for 2 h., to get the desired clear gel base. Regarding BRU loaded gel preparation, primarily; the drug was dissolved in ethanol then mixed with the prepared HPMC gel, the final concentration of the gel was 50 mg Bru in 20 g gel. 

### 3.6. Characterization of the Developed BRU Loaded Ethosomal Gel

#### 3.6.1. Visual Examination

All fabricated preparations including BRU loaded gel and BRU loaded ethosomal gel were inspected visually for homogeneity. 

#### 3.6.2. pH Measurement

Evaluating pH of the formulations were carried out by means of calibrated pH meter (MW802, Milwaukee Instruments, Szeged, Hungary) in order to insure weather the formulation irritant or not. 

#### 3.6.3. Spreadability

500 mg of gel or ethosomal gel was added between two slides and certain weight placed over them for 1 min. The formulation would spread in between the slides and the diameter of the spreading area was calculated [54]. 

#### 3.6.4. Viscosity

Brookfield viscometer (DV-II+ Pro, New York, NY, USA) was utilized to determine viscosity of BRU loaded gel and BRU loaded ethosomal gel formulation at room temperature [55].

#### 3.6.5. Morphological Evaluation

In concern with the morphology of the preparation, scanning electron microscopy (SEM), (JSM-6390LA, JEOL, Tokyo, Japan) was helpful in determine the structure of the optimized ethosomal gel formulation. Applying different magnifications (1000 to 95,000) and under vacuum, the sample was tested at 5 kv [56].

### 3.7. In-Vitro Release Experiment

The percentage of BRU released from BRU loaded gel, BRU loaded ethosome and BRU loaded ethosomal gel was determined and compared to that released from free BRU suspension using ERWEKA dissolution system (ERWEKA, GmbH, Heusenstamm, Germany). Glass tubes of the examined formulation (equivalent to 5 mg BRU) covered with cellophane membrane (MWCO 2000–15,000) from one end were placed into 750 mL PBS 7.4 kept at 37 ± 0.5 °C and. The apparatus rotated at 50 rpm and samples were withdrawn at definite time intervals up to 12 h and analyzed at λmax 264 nm. 

### 3.8. Cell Line

Melanoma cancer cells A375 were attained from American Type Culture Collection, (ATCC, Manassas, VA, USA). A375 cells were cultured in RBMI medium, supplemented with 10% heat- inactive (FBS) and augmented with 1% penicillin, 1% streptomycin and 4 mmol/L l-glutamine, using CO_2_ and incubated at 37 °C.

### 3.9. In-Vitro Cytotoxicity

The cytotoxic activity of BRU in all formulations including free BRU, the optimized ethosomal formulation and blank ethosome was examined on A375 cell lines by MTT assay [57]. Primarily, 3000 cells per well were seeded in to a 96-well plate and treated with fixed concentrations of BRU free drug, blank ethosome, BRU loaded gel and optimized BRU loaded ethosomal gel for 48 h. Afterward, MTT dye was added to check the cytotoxicity in each well in the and incubated for 4 h. The supernatant was detached, followed by adding DMSO to each well and were shaken for 10 min, then the absorbance was estimated at 570 nm [58].

### 3.10. Statistics

All experimental data were confirmed as mean ± SD related to three independent experiments at least. Data were compared to each other and statistically calculated using A one-way analysis of variance (ANOVA) was utilized to compare data from each other through SPSS statistics software, version 9 (IBM Corporation, Armonk, NY, USA). If *p* < 0.05, the difference would be verified as statistically significant.

## 4. Conclusions

In the current study, BRU loaded into optimized ethosomal formulation was well designed using response surface methodology and incorporated in to an HPMC gel base. The developed BRU loaded ethosomal gel demonstrated suitable vesicular size in nano scale with adequate physical characteristics, appropriate encapsulation efficiency accompanied with optimum flux. The in-vitro release of BRU was greatly affected when embedded in to ethosomal gel. Further, the flux through rat skin was significantly enhanced when being in ethosomal form. These findings revealed that the newly fabricated ethosomal gel could be a better alternative to conventional gel when supplied via the transdermal application. In addition, ethosome could be considered as a probable therapy for skin cancer, which would be further proved using in-vivo investigations to emphasize the efficiency of these preparations. 

## Figures and Tables

**Table 1:** Composition of various ethosomal formulations based on the experimental design and the resultant values of observed response.

Formula	Independent Variables		Dependent Response			PDI
	X_1_ (%)	X_2_ (%)	Y_1_ (nm)	Y_2_ (%)	Y_3_ (µg/cm^2^·h)	
F1	0.39	7.5	152 ± 2.5	61.4 ± 1.4	0.49 ± 0.025	0.250
F2	0.5	10	142 ± 1.7	74 ± 2.6	0.55 ± 0.035	0.230
F3	0.75	7.5	154 ± 2.0	66 ± 1.7	0.46 ± 0.04	0.311
F4	0.75	7.5	166 ± 1.5	70.2 ± 1.9	0.45 ± 0.035	0.290
F5	1	10	150 ± 1.4	76.3 ± 1.3	0.51 ± 0.04	0.271
F6	0.5	5	180 ± 2.1	55.8 ± 2.2	0.41 ± 0.05	0.321
F7	1	5	192 ± 2.0	58.9 ± 2.4	0.37 ± 0.031	0.401
F8	0.75	7.5	160 ± 1.6	68.6 ± 1.7	0.44 ± 0.038	0.304
F9	0.75	7.5	168 ± 1.8	71.5 ± 2.5	0.47 ± 0.035	0.281
F10	0.75	11.0	118 ± 1.5	77 ± 1.2	0.59 ± 0.04	0.214
F11	1.10	7.5	180 ± 2.5	73.5 ± 1.6	0.42 ± 0.038	0.282
F12	0.75	3.96	218 ± 3.0	50.2 ± 1.8	0.33 ± 0.015	0.336

X_1_: phospholipid concentration; X_2_: ethanol concentration; Y_1_ vesicular size; Y_2_: encapsulation efficiency; Y_3_: flux.

**Table 2:** Statistical and regression analysis results for all responses.

Source	Y_1_		Y_2_		Y_3_	
	F-Value	*p*-Value	F-Value	*p*-Value	F-Value	*p*-Value
Model	42.04	<0.0001 *	42.74	<0.0001 *	160.61	<0.0001 *
X_1_	5.68	0.0410 *	9.59	0.0241 *	22.79	0.0010 *
X_2_	78.39	<0.0001 *	0.2856	<0.0001 *	298.4	<0.0001 *
Lack of Fit	2.43	0.2489	1.81	0.3349	1.08	0.5160
R^2^ analysis						
R^2^	0.9033		0.9047		0.9727	
Adjusted R^2^	0.8818		0.8836		0.9667	
Predicted R^2^	0.8052		0.8310		0.9483	
Adequate Precision	17.707		17.681		34.550	
Model	Linear		Linear		Linear	
Remark	Suggested		Suggested		Suggested	

X_1_: phospholipid concentration; X_2_: ethanol concentration; Y_1_: vesicular size; Y_2_: encapsulation efficiency; Y_3_: flux; *: significant.

**Table 3:** Predicted and experimental value of response at the optimized conditions.

Dependent Variables	Symbol	Constraints
Vesicular size (nm)	Y_1_	Minimize
EE (%)	Y_2_	Maximize
Flux (%)	Y_3_	Maximize
Response	Predicted values	Experimental values
Y_1_ (nm)	140 ± 1.9	145.6 ± 2.3
Y_2_ (%)	71.1 ± 2.4	72.9 ± 2.1
Y_3_ (µg/cm^2^·h)	0.531 ± 0.04	0.513 ± 0.03

**Table 4:** Characterization of BRU loaded gel and ethosomal gel formulations.

Parameters	BRU Loaded Gel	BRU Loaded Ethosomal Gel
Visual examination	Smooth and homogenous	Smooth and homogenous
pH	5.8 ± 0.2	6.0 ± 0.3
Viscosity (cP)	4840 ± 375	4416 ± 277
Spreadability (mm)	41.7 ± 2.2	35.5 ± 0.7 *

Values are expressed as the mean ± SD (*n* = 3). * *p* < 0.05 compared to BRU gel.

**Table 5:** Special parameters related to ex vivo investigation from BRU formulations.

Formula	SSTF µg/cm^2^·h	ER
BRU suspension	0.212 ± 0.01	1
BRU gel	0.325 ± 0.027 *	1.53 ± 0.126 *
BRU ethosome	0.513 ± 0.03 *^,#^	2.42 ± 0.12 *^,#^
BRU ethosomal gel	0.40 ± 0.03 *^,#,^^■^	1.89 ± 0.12 *^,#,^^■^

Values are stated as mean ± SD (*n* = 3). * *p* < 0.05 when compared to BRU suspension. # *p* < 0.05 compared to BRU gel and ■ *p* < 0.05 compared to BRU ethosome.

**Table 6:** Independent variables, level of variation and the dependent variables.

Independent Variable	Symbol	Level of Variation		
		−1	0	+1
Phospholipid (%)	X_1_	0.5	0.75	1
Ethanol (%)	X_2_	5	7.5	10

## Data Availability

The data presented in this study are available on request from the corresponding author.

## Sample Availability

Not available.
